# Supplementary material for: Emotional Bias among Individuals at Risk for Seasonal Affective Disorder—An EEG Study during Remission in Summer
Source: Brain Sci. 2023 Dec 20;14(1):2. doi: 10.3390/brainsci14010002 (PMC10813094; doi:10.3390/brainsci14010002)
Supplement: Supplementary file 1 [file brainsci-14-00002-s001.zip › S1-brainsci-2756414-supplementary.pdf]

brain sciences

MDPI

Article Supplementary Section

Emotional bias among individuals at risk for seasonal affective disorder – an EEG study during remission in summer.

Dagný Theódórsdóttir[1] and Yvonne Höller [1,*]

[1] Faculty of Psychology, University of Akureyri, Akureyri, Iceland;
* Correspondence: yvonne@unak.is;

1. Statistics

All statistical analysis was carried out with R/R-Studio [1].

The supplementary analysis was conducted to control for the effect of age, since the group with elevated seasonality scores was significantly younger than the group with low seasonality scores. To this end, we first grouped individuals into a younger group of up to 50 years, and an older group of 50 years and older. This grouping is in line with a previous publication that showed that EEG responses in relation to seasonality interact with age [2]. According to this grouping, only three participants ended up in the high-seasonality and older group. Since this group size is too small to include the factor of age in a meaningful way into the ANOVA, we performed the same analysis as in the manuscript by excluding all participants who were older than 50 years.

Thus, EEG data was re-analyzed with the same semi-parametric repeated-measures ANOVA as the main analysis (MANOVA.RM). We conducted the analysis separately for the learning and recognition condition. For learning, we analyzed only the early time-window (100–300 ms), to address a potential attentional bias. For recognition, we included an additional repeated-measures factor time-window with the early (100–300 ms) and late (400–800 ms) time-windows as factor levels to differentiate between early attentional and late memory effects. Furthermore, for recognition we included the repeated-measures factor condition (old vs. new). In both analyses, we included the between-subject factor seasonality (low, high), and within-subject factors valence (negative, neutral, positive), and hemisphere (left, right).

2. Results

Table S1 shows the results of the semi-parametric repeated-measures ANOVA for the learning condition. None of the main effects or interactions were significant in the learning condition during the early (100–300 ms) time-window.

Table S1. Results of the semi-parametric repeated-measures ANOVA for learning, with between-subject factor seasonality (low, high), and within-subject factors valence (negative, neutral, positive), and hemisphere (left, right).

**Factor or Interaction**	*F*	*df*	*p*	*res.p*[1]
seasonality	0.38	1, 149.96	0.539	0.566
valence	1.66	1.97, Inf	0.190	0.182
hemisphere	2.99	1, Inf	0.084	0.096
seasonality x valence	0.85	1.97, Inf	0.426	0.419
seasonality x hemisphere	1.33	1, Inf	0.249	0.275
valence x hemisphere	1.83	1.97, Inf	0.160	0.170
seasonality x valence x hemisphere	0.53	1.97, Inf	0.586	0.577

[1] Resampling p-value obtained with parametric resampling and 1000 repetitions.

Table S2 shows the results of the semi-parametric repeated-measures ANOVA for the recognition condition.

Table S2. Results of the semi-parametric repeated-measures ANOVA for recognition, with between-subject factor seasonality (low, high) and within-subject factors valence (negative, neutral, positive), and hemisphere (left, right), time-window (100–300 ms, 400–800 ms), and condition (old, new).

**Factor or Interaction**	*F*	*df*	*p*	*res.p*[1]
seasonality	0.07	1, 136.41	0.791	0.811
valence	16.26	1.97, Inf	<0.001	<0.001
hemisphere	0.74	1, Inf	0.391	0.373
time-window	436.63	1, Inf	<0.001	<0.001
condition	0.52	1, Inf	0.471	0.476
seasonality x valence	2.65	1.97, Inf	0.072	0.083
seasonality x hemisphere	0.21	1, Inf	0.647	0.662
seasonality x time-window	2.20	1, Inf	0.138	0.035
seasonality x condition	1.67	1, Inf	0.196	0.210
valence x hemisphere	0.85	1.97, Inf	0.427	0.408
valence x time-window	5.00	1.95, Inf	0.007	0.013
valence x condition	4.75	1.77, Inf	0.011	0.015
hemisphere x time-window	0.36	1, Inf	0.549	0.571
hemisphere x condition	0.54	1, Inf	0.462	0.447
time-window x condition	0.41	1, Inf	0.524	0.517
seasonality x valence x hemisphere	0.32	1.97, Inf	0.720	0.732
seasonality x valence x time-window	3.16	1.95, Inf	0.044	0.052
seasonality x valence x condition	0.29	1.77, Inf	0.718	0.756
seasonality x hemisphere x time-window	0.01	1, Inf	0.914	0.922
seasonality x hemisphere x condition	0.17	1, Inf	0.682	0.678
seasonality x time-window x condition	0.41	1, Inf	0.520	0.504
valence x hemisphere x time-window	3.79	1.98, Inf	0.023	0.028
valence x hemisphere x condition	0.09	1.86, Inf	0.903	0.897
valence x time-window x condition	1.43	1.92, Inf	0.239	0.239
hemisphere x time-window x condition	0.19	1, Inf	0.662	0.660
seasonality x valence x hemisphere x time-window	0.06	1.98, Inf	0.940	0.947
seasonality x valence x hemisphere x condition	0.41	1.86, Inf	0.649	0.649
seasonality x valence x time-window x condition	0.40	1.92, Inf	0.662	0.674
seasonality x hemisphere x time-window x condition	0.48	1, Inf	0.490	0.495
valence x hemisphere x time-window x condition	0.09	1.94, Inf	.913	0.905
seasonality x valence x hemisphere x time-window x condition	0.55	1.94, Inf	00.569	0.561

[1] Resampling p-value obtained with parametric resampling and 1000 repetitions.

References

1. R Core Team R: A language and environment for statistical computing. *R Foundation for Statistical Computing* **2022**.

2. Höller, Y.; Jónsdóttir, S.T.; Hannesdóttir, A.H.; Ólafsson, R.P. EEG-responses to mood induction interact with seasonality and age. *Frontiers in psychiatry* **2022**, *13*, 950328, doi:10.3389/fpsyt.2022.950328.

Disclaimer/Publisher's Note: The statements, opinions and data contained in all publications are solely those of the individual author(s) and contributor(s) and not of MDPI and/or the editor(s). MDPI and/or the editor(s) disclaim

responsibility for any injury to people or property resulting from any ideas, methods, instructions or products referred to in the content.
